# Genetic Diversity and Utilization of Cultivated Eggplant Germplasm in Varietal Improvement

**DOI:** 10.3390/plants10081714

**Published:** 2021-08-20

**Authors:** Yusuff Oladosu, Mohd Y. Rafii, Fatai Arolu, Samuel Chibuike Chukwu, Monsuru Adekunle Salisu, Bolanle Amudalat Olaniyan, Ifeoluwa Kayode Fagbohun, Taoheed Kolawole Muftaudeen

**Affiliations:** 1 Institute of Tropical Agriculture and Food Security, Universiti Putra Malaysia (UPM), Serdang 43400, Malaysia; oladosuy@upm.edu.my (Y.O.); talk2fatty01@gmail.com (F.A.); chukwusamuel54@yahoo.com (S.C.C.); 2 Department of Crop Science, Faculty of Agriculture, Universiti Putra Malaysia (UPM), Serdang 43400, Malaysia; 3 Department of Agriculture, Faculty Technical and Vocational, Sultan Idris Education University, Tanjung Malim 35900, Malaysia; salisuadekunle@gmail.com; 4 Departments of Agronomy, Faculty of Agriculture and Forestry, University of Ibadan, Bodija 200284, Nigeria; abolaniyan@yahoo.com; 5 Department of Zoology, University of Lagos, Yaba 101017, Nigeria; fagbohunife@gmail.com; 6 Department of Biological Sciences, Faculty of Computing and Applied Sciences, Baze University, Abuja 900102, Nigeria; taoheed.muftaudeen@bazeuniversity.edu.ng

**Keywords:** diversity, genetic resources, morphological characterization, taxonomy, varietal improvement

## Abstract

Eggplant is the fifth economically most important vegetable in the Solanaceae family after tomato, potato, chili, and tobacco. Apart from the well-cultivated brinjal or aubergine eggplant (*Solanum melongena* L.), two other underutilized eggplant species, the African eggplant (*S. macrocarpon* L.) and the scarlet eggplant (*S. aethiopicum* L.), were also cultivated with local importance where the leaves and fruits are used for food and medicinal purposes. The major objectives of the eggplant breeding program are to improve fruit quality, increase yield performance through heterosis breeding, and introduce pest and disease resistances from wild relatives. Europe and Asia hold a wide collection of germplasm resources with significant potential for genetic improvement. While cultivated eggplant is susceptible to several fungi and bacteria, many wild relatives offer potential resistance to these pathogens. In this paper, we review the genetic resources and diversity of cultivated eggplant and its wild relatives. As a point of departure, we examine the economic importance, domestication, taxonomy characterization, and relationships of the crop and its wild relatives. The importance of evaluating and safeguarding wild relatives is highlighted, as crop wild relatives are highly underrepresented. A key section in this study is an overview dedicated to genetic resources, resistance to biotic and abiotic stresses, pre-breeding, and breeding for sustainable eggplant production.

## 1. Introduction

Eggplant (*Solanum melongena* L.), also known as brinjal in Southern Asia and aubergine in France and Britain, is the fifth economically most important vegetable in the Solanaceae family after tomato (*Solanum lycopersicum* L.), potato (*Solanum tuberosum* L.), chili (*Capsicum annuum* L.), and tobacco (*Nicotiana tabacum* L.). The fruit is prevalent in many essential diets across several local communities, especially in Africa, the subtropics (India, Bangladesh, Central America), Middle East, and Southeast Asia [1]. It is also cultivated in several warm temperate regions such as Southern USA and the Mediterranean [2]. Solanum is a large genus of over 1400 species, among which several members are poisonous to humans, such as *S. dulcamara* L. (the nightshades). Eggplant is considered an Old World crop that was domesticated in Africa, Asia, and Europe. In contrast, its relatives, such as tomato and potato, are New World crops with evidence of origination in South America [1]. The focus of this review will center on the Asian eggplant (*S. melongena*); However, two other Solanum species relative to the Asian eggplant viz; the Gboma/African eggplant (*S. macrocarpon* L.) and the Ethiopian/scarlet eggplant (*S. aethiopicum* L.) are partly discussed. The *S. macrocarpon* and *S. aethiopicum* are minor crops with local importance where the leaves and fruits are used for food and medicinal purposes. The similarities between these three species of eggplant have previously confused the taxonomic classification. However, they are comparatively far related within the genus [3].

Several non-exclusive concepts have been proposed regarding the origin of *S. melongena* L., also known as Asian eggplants. The most recent and reliable agreement is that the Middle Eastern/African species of *S. incanum* L. was intentionally transported into the Indo-China region, where the true wild progenitor of *S. insanum* L. evolved from which *S. melongena* was derived [3]. The first domesticated species are possibly represented by landraces and the comparatively small-fruited *S. ovigerum* where other cultivated types are derived. More recently, Meyer et al. [4] reported that eggplant was domesticated more than once according to molecular evidence. However, several arguments have emerged over eggplant domestication. For these reasons, the evolution and origin of eggplant present some important and interesting questions among researchers, which have been addressed using modern technologies. The domestication of Solanaceae members has been used as a model to study independent evolution trends. During domestication, selection is based on fruit size, shape, taste, and color, prevalent in other crops [5]. If the same genes are involved in multiple crops domestication processes, knowledge of these traits’ genetic basis can be transferred to other crops. For instance, eggplant and tomato exhibit several noticeable similarities in their domestication syndromes. Meanwhile, there is also evidence of similarities with pepper and potato in a set of traits, especially fruit size and shape in pepper and flower color, whereas this similarity was manifested in tuber for potato. When the first genetic maps of eggplant were produced, Doganlar et al. [6] reported a similar number of quantitative trait loci (QTL) in genomic regions of domesticated eggplant compared with other Solanaceae species in potato, chili, and tomato.

In recent times, little progress has been made in the utilization of eggplant wild relatives for the improvement of cultivated eggplants. Gramazio et al. [7] reported that one of the major hindrances in the utilization of wild species in the breeding program is the dearth of genome sequence information. This is, however, disappointing since the wild relatives are often the major sources of biotic and abiotic tolerance alleles [8]. The absence of a genome sequence for wild relatives also prevents the development of genome anchored markers required for effective trait transfer using marker-assisted selection. In practice, the introgression of a desirable gene from more distant eggplant relatives is quite challenging. Collonier et al. [9] reported that out of 19 wild species, only four produced fertile progenies when crossed with *S. melongena*, *S. aethiopicum*, *S. macrocarpon*, *S. linnaeanum*, and *S. incanum*. Nevertheless, there have been numerous successes in introgressing Fusarium wilt resistance from *S. aethiopicum* [10] and Verticillium wilt resistance from *S. linnaeanum* [11] into the cultivated eggplant. Lately, a large number of mapping populations from crosses between wild relatives and cultivated eggplant have been generated [12]. For years, the major objective in the eggplant breeding program has been to gain a better understanding of the genetic makeup of adaptive phenotypes in eggplant wild relatives. With the introduction of high-throughput sequencing, numerous wild species have been evaluated to generate the molecular markers necessary for candidate gene analysis, diversity analysis, and downstream genetic mapping.

While cultivated eggplant is susceptible to several fungi and bacteria, many of the wild relatives offer potential resistance to these pathogens. Therefore, efforts to understand the genetic basis for pathogenic resistance are extremely important. In this paper, we review the genetic resources and diversity of cultivated eggplant and its wild relatives. As a point of departure, we examine the economic importance, domestication, taxonomy characterization, and relationships of the crop and its wild relatives. The importance of evaluating and safeguarding wild relatives is highlighted, as crop wild relatives are highly underrepresented. A key section is an overview dedicated to genetic resources, resistance to biotic and abiotic stresses, pre-breeding, and breeding for sustainable eggplant production.

## 2. Economic Importance of Eggplant

**Figure 1:**
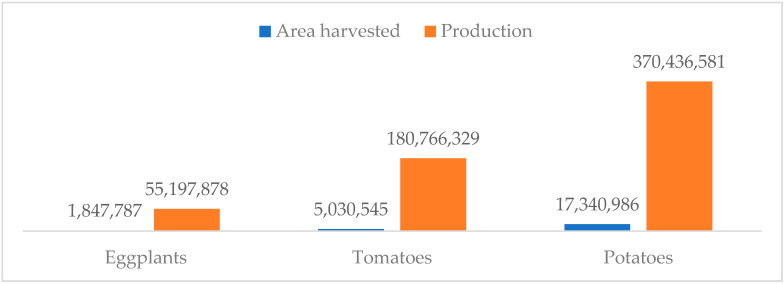
Harvested areas (hectares) and production (tonnes) of eggplant, potato, and tomato in 2019. Source: [13].

Among the Solanaceae family, eggplant is ranked third in harvested area and production after tomato and potato. In 2019, harvested area for tomatoes and potatoes worldwide was higher than that of eggplant by almost triple and a factor of 10, respectively (Figure 1). The greatest producers by countries are China (35.5 million) and India (12.6 million), followed by Egypt (1.2 million) and Turkey (0.8 million). Indonesia (0.5 million), the Philippines (0.2 million), and Sri Lanka (0.1 million) are also important eggplant producers in Southeast Asia (Table 1). The nutritional value of eggplant per 100 g according to the United States Department of Agriculture (USDA) was presented in Table 2. Eggplant’s fresh weight comprises 0.3 percent minerals, 0.3 percent fat, 1.3 percent fiber, 1.4 percent protein, 4 percent of various vitamins and carbohydrates (A and C), and 92.7 percent moisture. It is a fairly good source of potassium, phosphorus, calcium, iron, and the vitamin B group. Apart from its nutritional quality, eggplant has numerous health benefits in both orthodox and traditional medicine. Although eggplant is not known for its high health-promoting micronutrients, it has low calories and low fat, which make it valuable in diets. Remarkably, available literature suggested that eggplant is used as a medicine in different parts of the world for various illnesses. There is an increasing interest in using wild Solanum species in the pharmaceutical industry due to its rich content of different kinds of saponins and steroidal alkaloids. This may be a driving factor towards eggplant improvement and domestication in different parts of the world.

### 2.1. Bioactive Compounds of Eggplant

Agronomic properties such as fruit uniformity, increased yield, and resistance to biotic and abiotic stress has been the primary objective of traditional plant breeders. An increase in the global population, degradation of soil nutrients, and climate change have contributed to the declining quality and quantity of cultivated arable land; hence, disease resistance and improved fruit yield have been the major breeding priorities. In recent times, plant breeders have focused on enhancing chemical composition due to consumer awareness of the medicinal and nutritional value of vegetables and fruits [14]. Besides the accumulation of minerals that are important in human nutrition, plants produce numerous primary and secondary metabolites, which have a significant impact on human well-being. The primary metabolites are vitamins, lipids, proteins, and carbohydrates that directly involve plant developmental and physiological processes. In contrast, secondary metabolites are not important in plant rudimentary processes but play an essential role in protection against biotic and abiotic stresses. Though there is no specific classification of secondary metabolites, they are characterized as sulfur-containing compounds, nitrogen-containing alkaloids, terpenoids, and phenolics. Additionally, modern and traditional medicine relies on these phytochemicals as an essential source of pharmaceuticals and remedies for human illness. Therefore, metabolomic methods are becoming more significant in plant breeding.

#### 2.1.1. Phenolic

Eggplant is the best source of phenolic content, with the highest total among the Solanaceae family [15]. Several findings have reported a significant variation in phenolic content among eggplant germplasm. The highest content has been found in wild relatives of eggplant, including *Solanum incanum* L. and landraces [15,16]. Hence, landraces are another source of phenolic variations that can assist in selecting effective breeding programs. Similarly, the environment also constitutes a variation in total phenolic content. García-Salas et al. [17] reported differences in seasons where a significant decrease was observed from spring to summer, suggesting that high temperature has a negative effect. Therefore, this information can be used as a guiding principle to determine a suitable harvesting time for optimum phenolic content. In spite of the benefit of phenolics to humans and plants, it is also associated with disadvantages such as rapid fruit browning [18]. Generally, the browning of vegetables and fruits is a major problem in food industries as it causes great losses in quality during processing and postharvest storage. Enzymatic oxidation of phenolic compounds is the major cause of browning, and polyphenol oxidase is identified as the key enzyme in this degradation. Several researchers have characterized polyphenol oxidase activity under diverse environments using different cultivars and processing methods to reduce browning. Mishra et al. [18] reported significant browning inhibition through cutting using a sharp thin blade followed by immediately dipping in water for 10 min and drying at room temperature before packing. This method causes less cell death and physical injury; as a result, it reduces the leaching of polyphenol oxidase and phenolics, and thus less browning is achieved. Flavonoids are another significant eggplant phenolic compound with different concentrations in the leaves and fruits. The African eggplant (*Solanum anguivi*) has the highest concentration of flavonoids such as quercetin and rutin [19]. This, however, suggested that other species can be exploited due to potentially high flavonoids. The most predominant class of phenolic acid conjugates in eggplant is hydroxycinnamic acids, with their derivatives ranging from 8.6 to 13.6% of the total phenolic acid conjugates. There were also significant variations in hydroxycinnamic acids among eggplant genotypes [20]. Knowledge of the amount of hydroxycinnamic acid conjugates is important in eggplant breeding as it will help develop superior cultivars in hydroxycinnamic acid content and composition. Delphinidin glucosides derivatives of delphinidin anthocyanidin are one of the major anthocyanin occurring pigments in eggplant [17]. Anthocyanins are concentrated mostly in the fruit skin, ranging from 80 to 850 mg/kg peel with variations due to genetic and agronomic factors, temperature, light intensity, storage, and processing [21]. Raigón et al. [14] reported that conventionally grown eggplants had lower levels of total phenolics (382 mg/kg) compared to organically cultivated eggplants (498 mg/kg). However, Luthria et al. [22] observed no significant difference among organic (8900 mg/kg) and inorganic (9900 mg/kg) cultivated eggplant using an American variety ‘Blackbell’; hence, these results revealed that the content of phenolic compounds depends more on genotypes than on growing conditions.

#### 2.1.2. Carotenoids

Another active compound found in eggplant is carotenoids, although the amount in eggplant is less compared to other vegetables such as tomato and carrot. Carotenoids are ‘lipid-loving’ molecules that serve as accessory pigments in photosynthesis and protect the photosynthetic mechanism. The health benefits of these pigments have made them prevalent in dietary supplements and they are used as colorants in the food industry. Zeaxanthin and lutein have shown positive effects on age-related issues such as cataracts and macular degeneration [23]. The carotenoid content is affected by many factors, such as cooking treatment (frying, grilling, and cooking), postharvest conditions, plant stress, and the developmental stage. Zaro et al. [24] reported that the highest carotenoid levels were found at the early stages of fruit maturity, which gradually decrease during the ripening stage while postharvest storage at 0 °C protects against the deterioration of carotenoid levels. There is an increasing awareness of the potential of carotenoids in decreasing the risks of certain cancers due to their antioxidant properties. Hence, more studies are required to explore the potential of carotenoids in varieties of eggplant.

#### 2.1.3. Glycoalkaloids

Glycoalkaloids are nitrogen-containing steroidal glycosides found in eggplants and other Solanum members, including tomato and potato. Researchers showed glycoalkaloids play active roles in plants resistance against pathogens and pests [23]). α-solasonine and α-solamargine are the two main steroidal glycoalkaloids (SGAs) found in eggplant, and these SGAs have an anticarcinogenic effect in treating different types of cancers, such as basal cell carcinoma, osteosarcoma, lung cancer, liver cancer, leukemia, and gastric cancer [21]. In addition, literature has shown that the SGAs have an antiparasitic effect on *Trypanosoma cruzi*, *Leishmania amazonensis,* and *Leishmania mexicana* [21]. Though glycoalkaloids have beneficial effects as inhibitors of cancer cells, they are also toxic to humans and can cause death if injected in higher concentrations at 3 to 5 mg/kg body mass [25]. Therefore, the optimal levels for toxicity should be further studied.

### 2.2. Antioxidant Capacity of Eggplant

Reactive oxygen species (ROS) are dangerous entities produced by multiple cellular processes, which can be overproduced in reaction to different stimuli. The main source of ROS is incompletely processed electrons or oxygen produced by the electron transport chain (ETC) in the mitochondria [19]. Normal cells can maintain oxidative homeostasis due to various antioxidant systems that control ROS production through signaling and metabolic pathways changes. The free radical groups of ROS are highly disruptive and reactive to the chemical bonds of nearby molecules. Consequently, ROS are immediately recycled or neutralized after they are produced, this is mostly performed by antioxidants [21]. The ROS produced can damage DNA, lipids, and protein if not neutralized. This damage has been linked to cardiovascular and neurodegenerative diseases, as well as cancer. Furthermore, liver diseases are linked to ROS since the organ function as the recycling center [26]. In general, the human body synthesizes antioxidant enzymes; however, the antioxidant level is not enough to cope with the ROS produced. Hence, dietary sources of antioxidants are required. Eggplant is ranked among the top 10 of 120 antioxidant vegetables [27]. The total amount of these compounds ranges from 2664 to 8247 mmol trolox/kg depending on the variety, fruit shape, skin color, fruit size, postharvest storage temperature, and cooking methods [21].

## 3. Eggplant Origin and Domestication: First Insights

Many wild species of eggplant are related to *S. melongena* and the two other cultivated species and serve as sources of variations in breeding for adaptation to climate change and pest and disease resistance [28]. Vorontsova et al. [29] reported that the wild relatives are the most intricate and variable groups regarding their phylogenetic and taxonomic relationships [29]. Most of the wild eggplant relatives originating from Africa [3] are presented in Table 3. The wild types are almost inedible, spiny, bitter, small, and multi-seeded fruit. Based on biosystematics and crossing data, *S. melongena,* together with nine wild species, form the “eggplant complex”, which includes the cultivated brinjal eggplant and its closest wild relatives [30]. The gene pool concept was used to classify wild relatives based on their crossability with cultivated eggplant into tertiary, secondary, and primary gene pools [31]. The tertiary gene pool consists of distantly related species (e.g., *S. sisymbriifolium* Lam, *S. elaeagnifolium* Cav., and *S. torvum* Sw.,) used in breeding programs for their resistance characters which require specific cross-breeding techniques to succeed [32,33]. The secondary gene pool comprises many wild relatives (over 40) that are phylogenetically close to *S. melongena*. The success of the crosses, fertility, and viability of wild eggplants with cultivated types may be reduced. For instance, the interspecific hybridization derived is partly sterile due to reproductive obstacles such as *S. tomentosum* L., *S. linnaeanum* Hepper, and *S. dasyphyllum*, [33]. The primary gene pool of brinjal eggplant includes wild ancestor S. insanum and cultivated eggplant that can be crossed without difficulty to produce fertile and normal hybrids [32].

Phylogenic relationships between *S. insanum,* the wild progenitor for *S. melongena* and their closest African wild relative *S. incanum,* were recently clarified [30]. *S. dasyphyllum* and *S. anguivi* were confirmed as the wild progenitors of *S. macrocarpon* and *S. aethiopicum,* respectively. The three cultivated eggplants had a common and complex domestication event, as well as morphological changes associated with their domestication [37]. Similar seed, plant, and fruit traits were impacted in the same directions, although it seems that the domestication process is more advanced for *S. melongena* than the other two cultivated eggplants [37]. At the whole genome scale, the impact of domestication on tomato, pepper, and *S. melongena* has been shown to affect both gene expression and genetic architecture [37]. Hence, comparing the domestication signatures on *S. macrocarpon, S. aethiopicum*, and *S. melongena* genomes should bring further insights into the similarities and differences between the three cultivated eggplants.

The cultivation of small-fruited eggplant in China dates back to the 4th century, while evidence of cultivation in Africa indicated it began in the 9th century [38]. Though cultivated from prehistoric times, for many centuries, the growing of eggplant seems to be unknown to the Western World. This is evidenced by several African and Arabic names for eggplant and the lack of Roman and ancient Greek names, which indicated that this vegetable was introduced by Arabs to the Mediterranean in the late 7th century. The name Melongena, of Arabic origin, was given to one of the eggplant genotypes. Similarly, Avicenna, popularly known as Ibn Sina, “the father of modern medicine”, mentioned eggplant as a vegetable and medicinal plant. *S. macrocarpon* and *S. aethiopicum* are the most common and popular eggplants native to Africa, especially in Central and West Africa. However, the production of these crops remains relatively low, with limited information on the cultivated area and yield performance. West Africa is the center of diversity for these eggplants. Generally, eggplants are grown in Africa, mainly in small fields near villages and backyard gardens [38]. *S. macrocarpon* is widely cultivated in tropical America and Asia, while *S. aethiopicum* is popularly grown in South America.

The *S. aethiopicum* is a leafy and fruity vegetable that can be cooked or consumed raw. Its leaves can be consumed in the same way as spinach [38]. It is an herbaceous shrub with glabrous or hairy leaves and hermaphroditic flowers that can be cross or self-pollinated, which exist in clusters or as single flowers. The fruits are dark to light green, white, or blackish in color, with different tastes varying from bitter to sweet depending on the content of saponin, mostly in the case of oval-shaped cultivars. The fruit’s shape is oval, elongated-round, or round, with a grooved, smooth, or ribbed surface. At maturity, the fruits turn reddish-orange or red due to the high content of carotene.

The *S. macrocarpon* is widely cultivated for its glabrous and large leaves as a green vegetable. The fruits are large, with clasping calyx ranging from 2 to 6 cm in length and 3 to 10 cm in width. They are green or green-white, cream, white, and sub-spherical. The fruits are sweeter compared to *S. aethiopicum* and are more preferred. At the full maturity stage, the fruits turn brown, orange, or yellow with a ruptured surface [38].

*S. melongena* is characterized as a tall plant with spiny, large leaves. The flower is andromonoecy and in clusters. Furthermore, the fruit is bitter, green, and small in size with hard flesh and thick skin. The fruit color varies from dark to light purple, with some sub-species being white, green, or almost black. Its size ranges between 4 to 45 cm in length and 2 to 35 cm in width at different weights and shapes ranging between 15 g to 1.5 kg. The fruits are in clusters or single fruit set with up to 5 fruits per cluster. At full physiological maturity, the fruits become yellow, red, or brown [38]. Extensive human selection, mutation, domestication, hybridization, and natural inter-crossing have brought about genetic diversity among cultivated eggplants globally. Cultivar differences are mainly concerned with the agronomic and fruit qualities such as shape, color, fruit length, earliness, yield, chemical composition, and environmental requirements. At present, eggplant is the third most important crop from the Solanaceae family after potato and tomato.

## 4. Global Germplasm Collection and Conservation

Eggplant’s genetic resources have been collected systematically in some Asian and European countries. The Global Biodiversity Information Facility (GBIF) has recorded over 1.5 million occurrences of Solanum which could be biodiversity records, herbarium samples, or natural populations [34]. The largest cluster of *S. melongena* was recorded in India, with over 5000 of the total record of 21,000 globally (Table 3). Other predominant clusters are in Spain, Southeast Asia, and Turkey, while the major global occurrence of *S. macrocarpon* and *S. aethiopicum* was in West Africa with a total of 1365 and 4230, respectively [34]. The Global Gateway to Genetic Resources had a total of 95 accessions of *S. macrocarpon*, 590 of *S. aethiopicum,* and 4056 of *S. melongena*, as reported by GENESYS [35]. The Asian Vegetable Research and Development Center (AVRDC) Shanhua, Taiwan, is also one of the largest genebank holders of the three cultivated eggplants with 42 accessions of *S. macrocarpon,* 60 of *S. aethiopicum,* and 2256 of *S. melongena*, as reported by AVGRIS [36], followed by the Plant Genetic Resources Conservation Unit at the University of Georgia. The USDA-ARS had over 800 accessions, including 4 of *S. macrocarpon*, 60 of *S. aethiopicum,* and 770 accessions of *S. melongena* under the Germplasm Resources Information Network (GRIN) database. Gangopadhyay et al. [39] reported an estimated 1800 eggplant landraces, cultivars, and wild species in India collected by the National Bureau of Plant Genetic Resources, NBPGR, New Delhi. Similarly, Mao et al. [40] reported close to 2000 eggplant genotypes in China by the Institute of Vegetable Crops, IVC, Nanjing and Hangshu; The National Institute of Agrobiological Sciences, NIAS, Tsukuba in Japan had 31 accessions of *S. aethiopicum* and 561 of *S. melongena*; The Vavilov Research Institute of Plant Industry, St. Petersburg, Russia recorded 238 accessions of *S. melongena* [41]; and the National Gene Bank of China (NGBC) had 1300 accessions of *S. melongena*. There are some reports of collected germplasm resources in the Middle East [42], Southeast Asia [43], Africa [44], and Indonesia [45]. A more comprehensive database of eggplant-related germplasm estimating around 6000 accessions of *S. melongena, S. aethiopicum,* and *S. macrocarpon* was compiled by the Eggplant Genetic Resources Network (EGGNET project) in Europe project, a network of private and public sector researchers from the UK, Germany, Greece, Spain, Italy, France, and the Netherlands. This database is currently managed by the European Cooperative Programme for Plant Genetic Resources (ECPGR), Nijmegen, Netherlands. Castañeda-Álvarez et al. [46] reported that eggplants were among the crops whose wild gene pools are highly underrepresented. Undeniably, there is a need for conducting conservation actions and collection missions for wild eggplant relatives.

## 5. Strengthening Interdisciplinary Collaborations for Management and Utilization of Germplasm

In the 1970s, very few *S. melongena* germplasm collections existed in public institutions. Private breeders worked mostly with local material for their national market. The International Board for Plant Genetic Resources’ inputs was created in 1971 (now Bioversity International) and worldwide national initiatives. Many public collections have been progressively assembled for saving local material endangered by the intensification of horticulture and research purposes. According to online databases, *S. melongena* germplasm is relatively well-represented in genebanks worldwide. For the two indigenous African eggplants, *S. aethiopicum* and *S. macrocarpon*, efforts have been developed to protect their diversity and complete ex situ collections. Wild Asian species have been partly collected in the last decade via national and collaborative Asian projects, but apart from the World Vegetable Center (formerly AVRDC), little is known about these collections [47].

Bi-national projects (France and the UK in the 1990s) and the EU ESIN project (1993–1994) were set up as the first collaborations among experts of complementary disciplines ranging from botany and taxonomy to germplasm collections and genetics. A few years later, and within the framework of French and Dutch national agreements, vegetable breeding companies were connected to eggplant management and related species germplasm held by public institutions [47]. These converging forces were further integrated at the European scale within the EGGNET project. The challenges the eggplant community is facing nowadays invites further strengthening and widening of collaborations for at least three main reasons:

First, the large number of species related to cultivated eggplants is both an outstanding reserve of genes for breeders and a burden for germplasm holders, the supervision of which requires close collaboration with taxonomists [48]. Living collections of wild eggplant relatives are incomplete, both in terms of species and accessions per species, and their maintenance suffers from insufficient knowledge of each species’ biological peculiarities. Hence, there is a need to complete the collections with wild material and upgrade management in terms of seed production and maintenance of the accessions’ original genetic integrity.

Furthermore, access to wide germplasm resources is necessary to optimize the use of the powerful tools created by fast-evolving genomics and bioinformatics. Quantification and structuration of genetic and phenotypic diversity, limited for decades to a handful of species and accessions, is now accessible at whole collection and genome scales, as ambitioned by the EU G2PSol project (2016–2021). Hence, joint efforts among genebanks within and outside Europe are more imperative than ever to identify the strengths and weaknesses of the different collections and increase accessions for research and breeding.

Lastly, the exploration of phenotypic diversity for traits of interest within *S. melongena* and related species has been limited so far to a narrow range of accessions and traits and is clearly a bottleneck on future research efforts. Increased knowledge of germplasm-wide diversity is indispensable, particularly for resistance or resilience to biotic and abiotic stresses that are expected to increase in our changing climate. Phenotyping methods must also gain precision by intimate dissection of complex traits to identify their key regulatory genes and QTL networks.

## 6. Characterization of Eggplant Diversity

The morpho-physiological evaluation and characterization of available germplasm for targeted traits are major factors in the eggplant breeding program. These evaluations are essential for the sustainability and management of genetic resources. The major characterization involves measuring the plant traits that can be observed through simple visual observation at different growth stages such as the germination and seedling phase, vegetative stage, inflorescence descriptors, and maturity stage. Secondary morphological characterization deals with further complicated agronomic important traits such as biochemical properties, yield potential, fruit set, and pest and disease resistance [49]. These morpho-physiological descriptors allow easy and quick discrimination between phenotypes traits which are generally highly heritable traits and are equally influenced by changes in environmental conditions. The internationally accepted morphological descriptors for *S. macrocarpon, S. aethiopicum,* and *S. melongena* have been developed by the International Board for Plant Genetic Resources [50] and the World Vegetable Centre [36], which includes complete descriptions of important qualitative and quantitative traits illustrated either in arbitrary or metric scales (Table 4). The collection of eggplant germplasm has been evaluated generally for agronomic and morphological characters [2] revealing wide genetic variability in biochemical properties (antioxidant, alkaloids, anthocyanin, tannin, flavonoids, phenol, fruit bitterness), physiology (water use efficiency, flowering behavior) and plant morphology (yield potentials, fruit size, shape, and color, prickliness, hairiness, vigor, and plant growth habit) [9]. The most distinctive quality traits between wild relatives and cultivated Solanum species are fruit size, shape, and color [51].

The molecular diversity of wild and cultivated eggplant has been evaluated by several researchers to determine the genetic relationship for germplasm conservation and serve as a guide in the breeding program towards the development of superior lines. Different molecular markers have been used, including sequence-related amplified polymorphism (SRAP), inter-simple sequence repeat (ISSR), random amplified polymorphic DNA (RAPD), simple sequence repeat (SSR), allozymes, amplified fragment length polymorphism (AFLP), and chloroplast DNA markers. Several research studies have emphasized the superiority of molecular markers over morphological characterization in assessing the relatedness and diversity among eggplant species. Polignano et al. [52] evaluated 98 accessions of Asian eggplant, *S. melongena,* and two other Solanum species *viz*; African eggplants *S. macrocarpon* L. and the Ethiopian eggplant *S. aethiopicum* L. using 16 morphological traits. The results show that considerable diversity exists both between and within species. Based on cluster analysis, the accessions were clustered into three unrelated groups to the taxonomy classification of an accession (population, cultivar, subspecies, botanical, or variety group). This, however, revealed that morphological characterization is not a good predictor in genetic diversity assessment. Hence, the author suggests the use of molecular data as a better alternative for categorizing germplasm collections. Random amplified polymorphic DNA (RAPD) and allozyme data suggest that despite the fact that *S. incanum* (cultivated eggplant wild progenitor), *S. insanum* (weedy types), and *S. melongena* are morphologically different, these species are related at the genetic level [53]. While there is significant diversity between the wild and cultivated eggplants, the author argues that the designation differences are meaningless. Similarly, chloroplast DNA analysis of *S. melongena* and related species using restriction fragment length polymorphism (RFLP) revealed that taxonomic relationships based on morphological characters are, to a certain extent, unreliable [54]. However, similar clustering patterns have been obtained from both phenotypic data and molecular data (RAPD and AFLP markers) in comparative analyses of eggplant accessions (landraces, cultivars, and wild types) [42,55].

Furthermore, both molecular and morphological data were significantly useful in accurately classifying earlier mis-named and unnamed lines [55]. Hence, this suggested that morphological characterization is still more relevant in phylogenetic analysis of these taxonomically unclear plant groups. Evidently, the choice of accession and markers used will determine the conclusion drawn from the molecular diversity analysis. For example, while low genetic variability was observed within *S. melongena* using microsatellite markers [56], significant variations were observed within *S. melongena* and among related species using genic SSRs [57], RAPD [58], SRAP [59], and SSR [60]. Tiwari et al., 2009 and Isshiki et al., 2008 developed highly discriminatory RAPD and ISSR markers used for cultivar fingerprinting, and these markers were effective in revealing the phylogenetic relationship. Irrespective of the type of molecular marker used, it was observed that morphologically diverse cultivated eggplant has a narrow genetic background as compared with related species [57]. Muñoz-Falcón et al. [61] assessed the variability in landraces and commercial (hybrid and non-hybrid) eggplant within black-fruited accessions of *S. melongena* using morphological traits and polymorphic SSR markers. It was uncovered that higher genotypic and phenotypic diversity was observed among landraces as compared to commercial cultivars. Additionally, the hybrid’s accessions were observed to share a very limited gene pool. Hence, through breeding and efforts, domestication served as a diversity constraint within eggplants, while the non-hybrid and landrace varieties are still potentially valuable sources of heterozygosity for modern improved cultivars.

## 7. Classical Genetics and Traditional Breeding

Research efforts were concentrated on *S. lycopersicum* (tomato) as an essential model in early classical mapping, whereas *S. melongena* was ignored in this regard. Aside from anthocyanin accumulation, there is a dearth of phenotypic trait mapping in eggplant. Like other crop species, the quantitative nature of important agronomic traits made it difficult to conduct studies on inheritance in eggplant. Upon introducing molecular linkage maps and the accompanying breakthrough in comparative genomics, hitherto concentrated efforts on tomatoes have broadened to include eggplant, pepper, and potato. The genome-wide characterization of eggplant via molecular mapping is vital to breeding efforts on several levels. With this, it became easier to analyze the inheritance of complex traits and cull undesirable genotypes from breeding populations via marker-assisted selection. In contrast, the screening of germplasm for important traits was achieved with relative efficiency [62].

The primary aim of eggplant breeders was to increase yield and improve harvest quality by incorporating disease and pest resistance into the crop. Another important objective of the eggplant breeding program was to increase its tolerance to abiotic stress. With the advent of heterosis in brinjal, there have been concerted efforts to develop hybrids with improved productivity from inbred lines. Therefore, the majority of the commercial varieties are F_1_ hybrids. Despite this, eggplant breeding is limited by the laborious process of producing hybrid seeds. The process of manual emasculation and pollination of the inbred parents is time consuming and uneconomical. As a result, attempts are ongoing to incorporate cytoplasmic male sterility (CMS) into breeding lines of eggplants. Apart from heterosis breeding, the grafting of *S. melongena* onto tomato (*S. lycopersicum and S. hirsutum*) and other related species such as *S. macrocarpon, S. torvum, S. incanum,* and *S. aethiopicum* led to a massive improvement in eggplant production [63]. Furthermore, grafting is employed to fortify the plant, especially in the susceptible brinja cultivars, to resist soil pathogens in order to increase their yield [63]. Various stresses threaten the traditional cultivation of eggplant, including abiotic (salinity, heat, cold, drought, flooding), insect pest (leafhopper, nematode, Spider mite, beetle, aphid), bacterial (bacterial wilt, halo blight, and Tan spot), fungal (blight, mildew, anthrancnose), and viral (mosaic virus). Among the diseases, bacterial wilt has forced the local farmers to abandon the cultivation of solanaceous crops such as chili, bell pepper, tomato, and brinjal in affected areas [64]. Similarly, fruit and shoot borer infestation wreak heavy yield losses in eggplant cultivated areas, and this pest is very difficult to manage or control. During disease outbreaks, farmers indiscriminately use spurious heavy chemicals with high residual effects, leading to environmental pollutions. The commonly cultivated varieties have little resistance to pest and disease incidence, whereas some of their wild relatives have shown adequate pest and disease resistance. This prompted breeders to research into fortifying the commercial varieties by incorporating resistant genes into them. However, much of the breakthrough of such activities largely depends on the eggplant genotype, the crossing direction, and the phylogenetic distance between the parents [65]. Different biotechnological and conventional techniques are employed to develop horticulturally superior and high-yielding varieties with resistance to abiotic and biotic stresses.

One of the main setbacks in the inclusion of resistant genes from wild eggplants into commercial cultivars is cross-species’ incompatibilities, which manifests as sterility in the interspecific hybrids. The sterility or low fertility condition often results from meiotic irregularities [66]. However, various tissue culture techniques *viz*., genetic transformation, protoplast fusion, somatic hybridization, and haploidization, have been successfully exploited in wild and cultivated species with numerous success stories (Table 5). Protoplast fusion is advantageous in overcoming the pre- and post-fertilization obstacles faced in conventional breeding methods. This technique has enabled the easy transfer of desirable agronomic characters that are sexually incompatible in eggplants. Somatic hybridization is a different technique for developing interspecific hybrids of *S. melongena* [67]. While hybrids developed via this method usually express the desired trait, it is not uncommon for them to show the tendency to become sterile. While research has shown that there are exceptions [67], the inclusion of somatic hybrids into breeding activities is usually limited by their tetraploid characteristic. However, Anther culture has been shown as vital to achieving diploidy in such lines [68,69]. As a general rule, in attempts to incorporate genes from wild species into cultivated germplasm, meiotic recombination needs to occur between homeologues of the two parental species. There are indications showing a chromosomal exchange between the hybrids of *S. melongena* and *S. aethiopicum* somatic hybrids [68,69], and these hybrids can be used to confer bacterial and Fusarium wilt resistances into eggplants.

Eggplant is a self-pollinated crop, and consequently, the breeding approaches for its improvement include heterosis breeding, backcrossing, a combination of pedigree and bulk methods, bulk method, pedigree method, and pure-line selection. Different wild types of eggplant that carry resistance against biotic stresses are extensively utilized for eggplant improvement (Table 6). In recent times, the focus of breeding exercise has been directed towards parthenocarpy i.e., the development of seedless fruits. Parthenocarpy enables the development of fruits under sub-optimal environmental conditions, such as sub- or supra-optimal humidity and temperature conditions, inadequate light, and intense precipitation. Meanwhile, research by Donzella et al. [82] indicated that seedless fruits have a better taste, palatable flesh, and undergo browning slower than the seeded fruits. Therefore, incorporating phytohormone treatments in flowering plants can bring about parthenocarpy. However, this practice is associated with high labor and capital costs, which makes it less desirable [83]. Therefore, a more sustainable method is the selection of the desired trait, and several parthenocarpic cultivars have been developed using this technique [84]. One of the major factors determining the quality of eggplant fruits is the Anthocyanin pigment due to its profound impact on color alongside its antioxidants. Renewed interest in plant secondary metabolites led to concerted efforts on the part of breeders to improve pigments and other compounds influencing nutritional quality. As a result, many new research studies are centered on the anthocyanin activities of several brinjal accessions and related species have been tested. According to the radical-scavenging profile of purified pigments, scientists suggest that lines having anthocyanin delphinidin 3-glucoside should rank at the top in terms of antioxidant properties [20].

Recently, more than 14 phenolic compounds, an essential group of antioxidants, have been found in eggplant accessions [20]. Studies were conducted in Spain, Taiwan, Italy, and Turkey to assess the phenolic content in several hybrids and varieties [85,86]. The results of the evaluation show varying contents of phenolics in the different lines. The study observed up to ~2-fold variation across different lines and the possibility of selecting materials with increased antioxidant content for breeding programs. Due to the role of phenolics in the oxidative browning of cut fruit, a negative quality trait, it is imperative to achieve a trade-off between these two traits. However, research on heritability in several brinjal varieties and landraces indicated that phenolics levels are only responsible for about 20 percent of the variability in browning incidence. This suggests the possibility of obtaining lines with a high phenolic content and a minimal level of oxidative browning [87]. Research shows that solamargine has anti-cancer properties [88,89], and both compounds have been reported as effective in combating parasitic trypanosomatids [90]. Among the tested *S. melongena* germplasms, the highest concentration of solamargine occurs in the pickling varieties [88]. However, the amount of these glycoalkaloids in some wild species (namely, *S. macrocarpon, S. sodomaeum, S. aethiopicum* and *S. integrifolium*) raised concern in relation to the possible toxicity that may result from the use of this species in *S. melongena* breeding activities [85,88]. It is imperative to evaluate the safety of these compounds before they are utilized in breeding programs.

## 8. Conclusions

In the past 50 years, eggplant breeding has turned from an exclusive field activity on the improvement of a few traits to a collective and highly technical process. From hundreds of progenies screened mostly for quantitative and qualitative yield, eggplant breeders now work on thousands of plants, with many varietal types grown year-round in several countries. Breeders have taken advantage of the synteny between eggplant, pepper, and tomato genomes to efficiently and effectively improve their breeding programs. However, there is more scope for future work in eggplants. Eggplant’s transcriptome sequencing is still in its initial phase, this will facilitate comparisons with other relative’s genome sequences, thus intensifying genetic information. With over a thousand markers developed in eggplant, it would be easier to explore genome regions and gene features that control quantitative and qualitative traits of interest. Focus on the improvement of nutritional composition, such as phenolic and anthocyanin, should be included as breeding objectives. In addition, the characterization of antioxidant activities among landraces, cultivars, and wild species will aid in the selection of the best germplasm for this important trait. As the nutritional benefits of eggplant have become widely recognized and higher yield cultivars are developed through genetic improvement, this crop will become a globally important vegetable crop. The food security of several nations depends on crops produced from genetic resources from other regions of the world; thus, plant genetic resources call for global attention due to their mutually beneficial role. Information on the characteristics and extent of genetic diversity within the crop species is important for a successful and efficient breeding program. It plays a major role in characterizing individual accessions and also as a guide in selecting parents for hybridizations. There is a vast range of genetic diversity in wild relatives compared to their cultivated counterparts, and this could be a useful source for resistance to biotic and abiotic stresses. At present, only a few wild relatives have been utilized in eggplant breeding, and the introgression of wild relatives to improve commercial varieties is yet to become popular. To achieve remarkable success in eggplant cultivation, efforts should be intensified on screening for abiotic and biotic stresses in wild relatives and the collection of important germplasm accessions for the production of superior eggplant varieties. This information, alongside genomics studies on the identification of genes and QTLs of agronomic importance and their associated markers, will go a long way to improve eggplant production. Apart from using modern technologies, the conventional breeding technique remains an important method of developing a new variety where wild relatives/ species are utilized. Meanwhile, somatic hybridization has been widely studied in eggplants as a means of overcoming limitations due to cross incompatibility where important agronomic traits from wild species are introgressed to the cultivated ones. Additionally, new opportunities are available to enrich the existing genetic pools by increasing cytoplasmic and nuclear variability due to the production of somatic hybrids. The assessment of genetic resources in brinjal, mainly based on phenotype, indicate several useful features in its wild relatives, however, there is a dearth of molecular markers for their characterization. These studies will contribute to the available genetic linkage map by accelerating the isolation and identification of genes and markers involved in resistance to pests and diseases that are useful for marker-assisted breeding. Further studies on genetic engineering in eggplant are required for biotic and abiotic resistance encoding genes. So far, only parthenocarpy and Bt endotoxin genes have been introduced successfully in eggplants. The cost of production may be reduced with the development of Bt eggplants along with minimal environmental and health effects. In the future, it is important to focus on the improvement of nutritional quality and the productivity of specific secondary metabolites. Although significant developments have been achieved through biotechnology, the improvement has not been exploited to its full potential. Hence, the information provided in this review will be of great importance for the utilization of eggplant wild relatives and the management of genetic resources in germplasm collections.

## Figures and Tables

**Table 1:** Production and area of eggplant in the world (2019).

Area	Area Harvested	Production	Area	Area Harvested	Production
China	782,998	35,590,700	Spain	3470	245,150
India	727,000	12,680,000	Mexico	2333	185,234
Egypt	43,818	1,180,240	Algeria	6047	184,145
Turkey	23,337	822,659	Syrian Arab Republic	8342	154,807
Iran	21,350	670,158	Iraq	8660	136,749
Indonesia	43,954	575,392	Sri Lanka	9877	134,863
Japan	8650	301,700	Kazakhstan	4812	108,065
Italy	9550	300,620	United States of America	2614	105,302
Philippines	21,819	249,890	Rest of the world	119,173	1,572,468
			Total	1,847,804	55,198,142

Source: [13].

**Table 2:** Nutritional value of eggplant per 100 g (USDA report 11209).

Nutrient (Unit)	Amount	Nutrient (Unit)	Amount
Proximates		Vitamins	
Sugars, total (g)	3.53	Vitamin K (Phylloquinone) (μg)	3.5
Fibre, total dietary (g)	3	Vitamin E (α-tocopherol) (mg)	0.3
Carbohydrate, (g)	5.88	Vitamin A, IU (IU)	23
Total lipid (fat) (g)	0.18	Vitamin A, RAE (μg)	1
Protein (g)	0.98	Folate, DFE (μg)	22
Energy (kcal)	25	Vitamin B6 (mg)	0.084
Water (g)	92.3	Niacin (mg)	0.649
Minerals		Riboflavin (mg)	0.037
Zinc, Zn (mg)	0.16	Thiamin (mg)	0.039
Sodium, Na (mg)	2	Vitamin C (mg)	2.2
Potassium, K (mg)	229	Lipids	
Phosphorus, P (mg)	24	Cholesterol (mg)	0
Magnesium, Mg (mg)	14	Fatty acids, total polysaturated (g)	0.076
Iron, Fe (mg)	0.23	Fatty acids, total monosaturated (g)	0.016
Calcium, Ca (mg)	9	Fatty acids, total saturated (g)	0.034

**Table 3:** Occurrences and conserved accessions in genebank of cultivated eggplant and wild relatives.

Scientific Name	GBIF [34]	GENESYS [35]	AVGRIS [36]
*Solanum nigrum*	211,385	44	20
*Solanum americanum*	27,624	43	189
*Solanum melongena*	21,852	4056	2256
*Solanum torvum*	12,775	115	39
*Solanum villosum*	11,590	48	17
*Solanum sisymbriifolium*	7054	4	10
*Solanum nigrescens*	4794	1	2
*Solanum aethiopicum*	4230	590	60
*Solanum anguivi*	4098	6	39
*Solanum anguivi*	4098	23	4
*Solanum seaforthianum*	3713	3	5
*Solanum linnaeanum*	3327	4	3
*Solanum linnaeanum*	3327	3	3
*Solanum capsicoides*	2638	1	1
*Solanum viarum*	2237	3	17
*Solanum incanum*	2008	28	3
*Solanum aculeatissimum*	1873	46	19
*Solanum violaceum*	1606	1	59
*Solanum scabrum*	1400	148	55
*Solanum macrocarpon*	1365	95	42
*Solanum lasiocarpum*	1076	31	34
*Solanum virginianum*	1032	1	3
*Solanum virginianum*	1032	3	3
*Solanum trilobatum*	207	10	7
*Solanum ferox*	150	11	8
*Solanum insanum*	110	11	16
Grand total	1,518,222	5204	2907

**Note**: Global Biodiversity Information Facility (GBIF), Global Gateway to Genetic Resources (GENESYS), AVRDC Vegetable Genetic Resources Information System (AVGRIS).

**Table 4:** Standard eggplants descriptors for seedlings, vegetative, inflorescence, seed, and fruits traits adapted from the International Board for Plant Genetic Resources [50] and the World Vegetable Centre [36].

Seedling Traits	Unit	Scale
Germination period	no.	Number of days from sowing to first seed germination
Cotyledonous leaf color	-	7 = Violet; 5 = Light violet; 3 = Green
Cotyledonous leaf length	mm	N = 10
Cotyledonous leaf width	mm	N = 10
Cotyledon length to width ratio	-	9 = Very high (>5.0); 7 = High (~3.5); 5 = Intermediate (~2.5); 3 = Low (~2.2); 1 = Very low (<2.0)
Vegetative Traits		
Plant breadth at flowering stage	cm	9 = Very strong (>130); 7 = Broad (~90); 5 = Intermediate; 3 = Narrow (~40); 1 = Very narrow (<30)
Plant height at flowering stage	cm	9 = Very tall (>150); 7 = Tall (~100); 5 = Intermediate (~60); 3 = Short (~30); 1 = Very short (<20)
Plant growth habit	-	7 = Prostrate; 5 = Intermediate; 3 = Upright; 1 = Very upright
Stem ridging	-	7 = Prominent; 5 = Intermediate; 3 = Shallow; 0 = Absent
Degree of stem pubescence	-	4 = Very many; 3 = Many; 2 = Intermediate; 1 = Few; 0 = Absent
Spines on stem	-	7 = Long; 5 = Intermediate; 3 = Short; 0 = Absent;
Number of primary branches per plant	no.	9 = Very strong (>30); 7 = Strong (~20); 5 = Intermediate (~10); 3 = Weak (~5); 1 = Very weak (~2)
Petiole length	mm	9 = Very long (>100); 7 = Long (~50); 5 = Intermediate (~30); 3 = Short (~10); 1 = Very short (<5); 0 = None
Petiole color	-	9 = Dark brown; 7 = Dark violet; 3 = Violet; 2 = Greenish violet; 1 = Green
Leaf blade length	cm	7 = Long (~30); 5 = Intermediate (~20); 3 = Short (~10)
Leaf blade width (maximum width)	cm	7 = Wide (~15); 5 = Intermediate (~10); 3 = Narrow (~5)
Leaf blade tip angle	-	9 = Very obtuse (>160°); 7 = Obtuse (~110°); 5 = Intermediate (~75°); 3 = Acute (~45°); 1 = Very acute (<15°)
Leaf blade lobes	-	9 = Very strong; 7 = Strong; 5 = Intermediate; 3 = Weak; 1 = Very weak
Leaf blade color (upper surface)	-	9 = Violet; 7 = Greenish violet; 5 = Dark green; 3 = Green; 1 = Light green
Leaf hairs (no. of hair per mm^2^ on lower surface of the leaf)	no.	9 = Very many (>200); 7 = Many (100–200); 5 = Intermediate (50–100); 3 = Few (20–50); 1 = Very few (<20)
Leaf prickles (no. of leaf prickles on upper surface of the leaf)	no.	9 = Very many (>20); 7 = Many (11–20); 5 = Intermediate (6–10); 3 = Few (3–5); 1 = Very few (1–2); 0 = None
Inflorescence Traits		
Flowering time	no.	Number of days from sowing until first flower opening
Sepal length	cm	N = 5
Petal length	cm	N = 5
Stamen length	cm	N = 5
Style Exsertion	-	7 = Exerted; 5 = Intermediate; 3 = Inserted
Pollen production	-	7 = High; 5 = Medium; 3 = Low; 0 = None
Relative style length	mm	7 = Long (~5); 5 = Intermediate (~3); 3 = Short (~1);
Corolla color	-	9 = Bluish violet; 7 = Light violet; 5 = Pale violet; 3 = White; 1 = Greenish white; 0 = Yellow
Seed Traits		
100 seeds weight	gm	-
Seed size (diameter)	mm	7 = Large (~4); 5 = Intermediate (~3); 3 = Small (~2)
Seed density	-	7 = Dense; 5 = Intermediate; 3 = Scarce
Number of seeds per fruit	-	9 = Very many (>500); 7 = Many (~300); 5 = Intermediate (~100); 3 = Few (~50); 1 = Very few (<10); 0 = None
Seed color	-	9 = Black; 6 = Brown black; 5 = Brown; 4 = Brownish yellow; 3 = Grey yellow; 2 = Light yellow; 1 = White
Fruit Traits		
Fruiting date	no.	Days to 50% mature fruits per plant
Fruit breadth (diameter at broadest part)	cm	9 = Very large (>10); 7 = Large (~5); 5 = Intermediate (~3); 3 = Small (~2); 1 = Very small (<1)
Fruit length (from base of calyx to tip of fruit)	cm	9 = Very long (>20); 7 = Long (~10); 5 = Intermediate (~5); 3 = Short (~2); 1 = Very short (<1)
Fruit length/breadth ratio	-	9 = Several times as long as broad; 8 = Three times as long as broad; 7 = Twice as long as broad; 5 = Slightly longer than broad; 3 = As long as broad; 1 = Broader than long
Fruit calyx prickles (N = 10)	no.	9 = Very many (>30); 7 = Many (~20); 5 = Intermediate (~10); 3 = Few (~5); 1 = Very few (<3); 0 = None
Fruit cross-section	-	9 = Very irregular; 7 = Many grooves (~8); 5 = Few grooves (~4); 3 = Elliptic, no grooves; 1 = Circular, no grooves
Fruit pedicel prickles	no.	9 = Very many (>30); 7 = Many (~20); 5 = Intermediate (~10); 3 = Few (~5); 1 = Very few (<3); 0 = None
Fruit pedicel thickness	mm	9 = Very thick (>10); 7 = Thick (~5); 5 = Intermediate (~3); 3 = Thin (~2); 1 = Very thin (<1)
Fruit pedicel length	mm	9 = Very long (~75); 7 = Long (~50); 5 = Intermediate (~25); 3 = Short (~10); 1 = Very short (<5)
Fruit color at commercial ripeness	-	9 = Black; 8 = Purple black; 7 = Purple; 6 = Lilac gray; 5 = Scarlet red; 4 = Fire red; 3 = Deep yellow; 2 = Milk white; 1 = Green
Fruit curvature	-	9 = U shaped; 8 = Sickle shaped; 7 = Snake shaped; 5 = Curved; 3 = Slightly curved; 1 = None
Fruit yield per plant	gm	9 = Very high (>5000); 7 = High (~2500); 5 = Intermediate (~1000); 3 = Low (~500); 1 = Very low (<250)
Fruit flesh density	-	9 = Very dense; 7 = Dense; 5 = Average density; 3 = Loose (crumbly); 1 = Very loose (spongy)
Fruit color at physiological ripeness	-	9 = Black; 8 = Light brown; 7 = Scarlet red; 6 = Poppy red; 5 = Fired red; 4 = Deep orange; 3 = Yellow orange; 2 = Deep yellow; 1 = Green
Fruit position	-	9 = Pendant; 7 = Semi-pendant; 5 = Horizontal; 3 = Semi-erect; 1 = Erect
Fruit apex shape	-	7 = Depressed; 5 = Rounded; 3 = Protruded
Varietal mixture condition	-	7 = Serious mixture; 5 = Medium mixture; 3 = Slight mixture; 0 = Pure
Fruit color distribution at commercial ripeness	-	7 = Striped; 5 = Netted; 3 = Mottled; 1 = Uniform
Fruit shape	-	7 = About 3/4 way from base to tip; 5 = About 1/2 way from base to tip; 3 = About 1/4 way from base to tip
Fruit flavor	-	7 = Sweet; 5 = Intermediate; 3 = Bitter
Relative fruit calyx length	mm	N = 10
Number of locules per fruit	no.	N = 10
Number of fruit per plant	no.	Total number of fruit per plant

**Table 5:** Development of somatic hybrids through protoplast fusion in eggplant.

Parents	Fusion	Hybrid Characteristics	Source
*S. melongena ×* *S. tuberosum*	Electrical	Introgression of bacterial wilt resistance to *Solanum tuberosum* from *Solanum melongena*	[70]
*S. integrifolium ×* *S. sanitwongsei*	UV	*Ralstonia solanacearum* Resistance	[67]
*S. melongena ×S. aethiopicum* gr. Aculeatum	Electrical	Fertile and fusarium wilt resistant	[71]
*S. melongena ×* *S. sanitwongsei*	polyethylene glycol	Fertile and bacterial wilt resistant	[72]
*S. melongena ×S. aethiopicum* gr. Aculeatum	Electrical	*Ralstonia solanacearum* Resistant and high-yielding	[73]
*S. melongena ×* *S. integrifolium*	-	Fertile and bacterial wilt resistant	[74]
*S. melongena ×* *S. nigrum*	Electrical	Sterile and atrazine resistant	[75]
*S. melongena ×* *S. torvum*	Electrical	Sterile hybrid resistance to nematodes and *Verticillium dahlia*	[76]
*S. melongena ×* *S. nigrum*	polyethylene glycol	Sterile and atrazine resistant	[77]
*S. melongena ×* *S. torvum*	polyethylene glycol	Sterile hybrid partial resistance to mites and resistance to Verticillium wilt	[78]
*S. melongena × S.* *khasianum*	Electrical	Sterile and *Leucinodes orbonalis* resistant	[79]
*Solanum melongena ×* *S. sisymbrifolium*	polyethylene glycol	Sterile hybrid resistant to mites and nematodes	[80]
*S. melongena ×* *S. torvum*	Electrical	*Ralstonia solanacearum* and *Verticillium dahlia* resistant	[81]

**Table 6:** Identified eggplant germplasms resistant to biotic stresses and useful traits for breeding.

Trait	Source	Reference
Powdery mildew (*Leveillula taurica)*	*S. pseudocapsicum, S. aviculare, S. aculeatissimum, S. linnaeanum,*	[91]
Phomopsis fruit rot (*Phomopsis vexans*)	*Solanum xanthocarpum, S nigrum, S gila, S indicum, S. khasianum, S sisymbrifolium*	[92]
Fusarium wilt (*Fusarium oxysporum* f. sp. *melongenae*)	*S integrifolium*	[93]
Spider mite (*Tetranychus urticae*)	*S. sisymbrifolium,* *S. pseudocapsium, S. mamosum, S. integrifolium, S. macrocarpon,*	[94]
Eggplant fruit and shoot borer (*Leucinodes orbonalis* Guenee)	*S. melongena:* VI047451; *S incanum; S integrifolium; S hispidum; S. khasianum*,	[95,96,97,98]
Root Knot Nematode (*Meloidogyne javanica*)	*S. melongena*: A-264-A; *S. torvum* ‘CNPH 610′	[99]
Leafhopper (*Amrasca devastans* Distant)	*S. melongena*: VI035835, VI035822, VI034971	[100]
Spotted beetles (*Epilachna vigintioctopunctata* Fabricius)	Shankar Vijay, Hissar Selection 14, Arka Shirish	[101]
Little leaf disease	*Solanum hispidum*; *S. melongena*: Nurki, Hisar Shyamal, H-10	[97,102]
Bacterial wilt (BW), (*Ralstonia solanacearum*)	BG 219; EG 203; BG 192; TS 3; *S. melongena*: TS90, TS87, TS69, TS47, VI034885 and TS3	[100,103]
Verticillium wilt (*Verticillium* spp.)	Skoutari, EMI, *S. linnaeanum*	[11,104]
Phytophthora blight (*Phytophthora capsici*)	PI413784	[105]
leaf mosaic virus	*S. hispidum*	[97]
aphids	*S. hispidum*	[97]
High antioxidant activity	*S. aethiopicum*: S00197, *S. melongena*: S00022, S00062	[86]
Early maturity	*S. melongena*: VI046110	[100]
High yielding	*S. melongena*: EG235, EG233, VI44067, VI047332, VI046097, VI037736, VI046110, VI047333, VI045551	[106]

## Data Availability

All data is presented within the article.
